# Climate drives intraspecific differentiation in the expression of growth-defence trade-offs in a long-lived pine species

**DOI:** 10.1038/s41598-020-67158-4

**Published:** 2020-06-29

**Authors:** Carla Vázquez-González, Luis Sampedro, Vicente Rozas, Rafael Zas

**Affiliations:** 1 Misión Biológica de Galicia - Consejo Superior de Investigaciones Científicas (MBG-CSIC), 36143 Pontevedra, Spain; 2 iuFOR-EiFAB, Campus Duques de Soria, Universidad de Valladolid, 42004 Soria, Spain; 3 Laboratorio de Dendrocronología y Cambio Global, Facultad de Ciencias Forestales y Recursos Naturales, Universidad Austral de Chile, Valdivia, Chile

**Keywords:** Genetic variation, Evolutionary ecology, Natural variation in plants, Plant ecology, Plant evolution, Plant stress responses, Secondary metabolism, Forest ecology

## Abstract

Intraspecific variation in plant defences is expected to be the result of adaptive and plastic responses to environmental conditions, where trade-offs between growth and defences are thought to play a key role shaping phenotypic patterns in defensive investment. Axial resin ducts are costly defensive structures that remain imprinted in the tree rings of conifers, therefore being a valuable proxy of defensive investment along the trees’ lifespan. We aimed to disentangle climate-driven adaptive clines and plastic responses to both spatial and temporal environmental variation in resin duct production, and to explore growth-defence trade-offs. To that aim, we applied dendrochronological procedures to quantify annual growth and resin duct production during a 31-year-period in a Mediterranean pine species, including trees from nine populations planted in two common gardens. Both genetic factors and plastic responses modulated annual resin duct production. However, we found no evidence of adaptive clines with climate gradients driving population differentiation. Our results revealed a marked physiological trade-off between growth and defences, where the slope of the trade-off was genetically variable and associated with climatic gradients. Our results help to enlighten the evolutionary patterns and genetic basis of defensive allocation within species, particularly revealing a key role of growth-defence trade-offs.

## Introduction

Plants protect against their enemies with an impressive array of chemical and anatomical defensive traits known to display large variation among and within species^1–3^. Particularly, understanding the origin and maintenance of intraspecific diversity patterns in plant defences has been a central question in ecology over the past half century^3–5^. On the one hand, studies in common gardens have shown that among population variation in defensive investment can be associated with environmental gradients at the origin of populations, suggesting patterns of local adaptation^6–9^. On the other hand, phenotypic plasticity can also play a major role modulating allocation to plant defence^10–12^.

Increasing biotic and abiotic stresses can compromise the future survival and performance of plant populations^13,14^. While migration of plant populations towards new environmentally optimum locations can be possible^15^, the speed and intensity of environmental changes may limit such responses^16^. In those cases, adaptive and plastic responses remain as crucial mechanisms to combat the negative impacts of increasing abiotic and biotic stresses on plant populations^16–19^. Environmental clines in plant functional traits inform about past adaptation processes^17,18^. Locally adapted populations may, however, suffer suboptimal conditions as their current environment is changing^20^. In those cases, phenotypic plasticity can buffer the negative impacts of environmental stress^21^. Furthermore, plasticity can also vary among populations, i.e. genotype by environment interactions (G × E), where reaction norms to environmental changes may not be homogenous across the species distribution ranges^22^. Disentangling the individual and interactive effects of adaptive and plastic responses in functional traits is therefore crucial to anticipate future performance of plant populations facing environmental changes.

A central paradigm in the field of plant defence research is that, because plant resources are limited, defensive investment may physiologically trade-off with other plant functions such as growth and reproduction^5,23^. Particularly, growth-defence trade-offs are thought to drive variation in defensive investment among and within species^4,24,25^. Several ecological theories have been proposed to explain allocation balance between growth and defence. For instance, the resource availability hypothesis (RAH)^26,27^ posits that resource-poor environments select for slow-growing, highly-defended plant species, as relative costs for replacing the tissues damaged by herbivores are higher under stressed conditions. Similarly, the growth-differentiation balance hypothesis (GDBH)^5^ states that shortage in any resource that limits growth but not photosynthetic activity leads to enhanced defence production promoting the expression of physiological trade-offs. The existence of evolutionary growth-defence constraints has been commonly identified among species^27^. However, much less is known about such patterns within species, particularly among populations, where studies often report neutral, negative and positive associations between growth and defences^3^. One potential explanation for these inconsistent patterns is that physiological growth-defence trade-offs are often noticed only under determinate environmental conditions^28,29^. Alternatively, if heterogeneous environmental conditions within a species distribution range select for different resource acquisition capabilities and growth-defence strategies, the strength and direction of growth-defence associations may differ among populations^3,24,30^. Intraspecific genetic variation in the expression of trade-offs might indeed mask negative correlations at a higher hierarchical level.

Pine trees (*Pinus* spp.) are large and long-lived organisms that occupy wide distribution ranges and are exposed to intense biotic and abiotic stresses through their lifespan. The defensive system of pine species particularly rely on the synthesis and storage of oleoresin, a complex viscous mixture of terpenoids that act as both chemical deterrent and mechanical barriers to herbivores^31,32^. Oleoresin is produced in specialized cells grouped to form complex tube-like structures known as resin ducts^32^. Density and conductive area of resin ducts have been positively associated with increased tree resistance to herbivores^33–36^. Axial resin ducts are imprinted in the annual growth rings in the secondary xylem. Interannual variation in axial resin duct production and growth in pine trees can be thus retrospectively quantified by standard dendrochronological procedures^37^. This approach provides an outstanding opportunity to link the temporal dimension of plastic responses to year-to-year climatic variability with the study of intraspecific variation in plant defences. Collecting multiple pairs of annually-resolved observations of secondary growth rate and defence production along the lifespan of trees allows us to test for the existence of physiological constraints between both plant functions. Furthermore, tree-ring analysis applied to mature trees in common gardens can help to elucidate the genetic basis of growth-defence trade-offs and to explore patterns of intraspecific genetic variation in the expression of such constraints.

**Figure 1:**
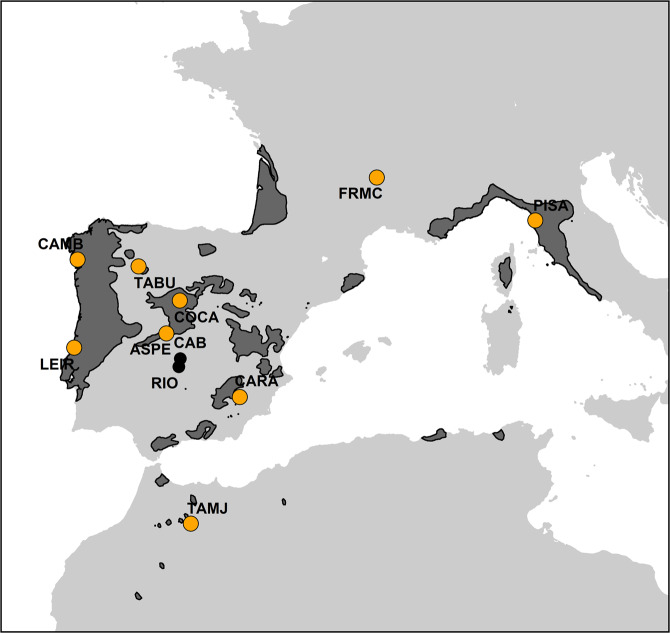
Distribution range of *Pinus pinaster* in Europe. Location of the two common gardens, i.e. test sites (black dots), Cabañeros (CAB) and Riofrío (RIO) in central Spain and the nine populations included in this study (orange dots) are shown. See population codes in Table S1. The map was generated using the *ggplot* function from the *ggplot2* package in *R* software version 3.6.0 (http://www.R-project.org/)^71,72^. The shapefile of the species distribution is publicly available at EUFORGEN (http://www.euforgen.org).

Here, we applied dendrochronological methods to investigate the individual and interactive effects of genetic variation and plasticity in anatomical defences (number and density of resin ducts) of *Pinus pinaster* trees from nine populations growing in two replicated common gardens (‘test sites’ hereafter) differing in environmental conditions (Fig. 1). Moreover, we investigated intraspecific patterns of covariation between growth and defences. Specifically, we investigated (i) genetic variation and plasticity, as well as their interaction (G × E), in the expression of anatomical defences, (ii) the extent to which genetic variation in anatomical defences is associated with climate at the population’s origin (i.e. local adaption), (iii) whether there are trade-offs between annual growth and annual production of anatomical defences and to what extent such constraints are variable among populations and between test sites, and finally, (iv) whether genetic variation in the expression of growth-defence trade-offs is driven by the climate at the population’s origin. Overall, this study builds towards a better understanding about how genetic adaptation, plasticity to local site conditions and individual plastic responses to year-to-year environmental variation might mediate evolutionary responses in growth-defences strategies.

## Results

### Genetic variation and phenotypic plasticity in anatomical defences

Average annual resin duct density across populations and test sites in the latewood (1.49 ± 0.002 RD mm^−2^ year^−1^; mean ± SE) was 6-fold greater than that in the earlywood (0.22 ± 0.006 RD mm^−2^ year^−1^), indicating that resin duct production relative to growth area was mainly restricted to the late growth season (Table S3).

Average annual number of resin ducts in the earlywood and latewood (but not in the total ring) significantly varied among pine populations (Table 1). Resin duct number in the earlywood ranged between 0.83 RD year^−1^ in CARA and 1.65 RD year^−1^ in COCA (Fig. S2a). Resin duct number in the latewood ranged between 1.51 RD year^−1^ in TABU and 2.54 RD year^−1^ in TAMJ (Fig. S2a). Resin duct density in the earlywood, latewood and the total ring also differed significantly among pine populations (Table 1). Resin duct density in the total ring varied between 0.45 RD mm^−2^ year^−1^ in CARA and 0.55 RD mm^−2^ year^−1^ in FRMC. Similarly, resin duct density in the earlywood ranged between 0.14 RD mm^−2^ year^−1^ in CARA and 0.28 RD mm^−2^ year^−1^ in FMRC (Fig S2b). Finally, resin duct density in the latewood ranged between 1.03 mm^−2^ year^−1^ in COCA and 1.55 RD mm^−2^ year^−1^ in TAMJ (Fig. S2b).

**Table 1:** Results of the mixed model analysis showing the across subjects effects of Population, Site and Population by Site interaction (Population × Site) on resin duct number (RD year^−1^) and density (RD mm^−2^ year^−1^) in the total ring, earlywood and latewood of *Pinus pinaster* trees from nine populations planted in two test sites.

	*NumDf, DenDf*	Total Ring		Earlywood		Latewood	
		*F*	*p*	*F*	*p*	*F*	*p*
**Resin duct number**							
***Across subjects***							
Population	8,156	1.95	0.056	4.90	**<0.001**	9.31	**<0.001**
Site	1,156	17.85	**<0.001**	37.60	**<0.001**	0.00	0.976
Population × Site	8,156	1.68	0.107	0.77	0.626	1.91	0.062
***Within Subjects***							
Year	30,4669	299.61	**<0.001**	302.49	**<0.001**	64.30	**<0.001**
Pop × Year	240,4669	2.92	**<0.001**	2.18	**<0.001**	2.18	**<0.001**
Site × Year	30,4669	25.61	**<0.001**	18.30	**<0.001**	13.12	**<0.001**
Pop × Site × Year	240,4669	2.05	**<0.001**	1.46	**<0.001**	1.35	**<0.001**
**Resin duct density**							
***Across subjects***							
Population	8,156	2.7	**0.008**	7.73	**<0.001**	5.33	**<0.001**
Site	1,156	6.6	**0.011**	49.90	**<0.001**	6.69	**0.011**
Population × Site	8,156	0.9	0.546	0.88	0.537	0.41	0.913
***Within Subjects***							
Year	30,4669	45.6	**<0.001**	91.14	**<0.001**	83.52	**<0.001**
Population × Year	240,4669	3.0	**<0.001**	1.84	**<0.001**	1.86	**<0.001**
Site × Year	30,4669	12.6	**<0.001**	13.11	**<0.001**	13.34	**<0.001**
Population × Site × Year	240,4669	2.00	**<0.001**	1.37	**<0.001**	1.25	**<0.001**

Within subjects, the effects of Year and its interaction with Population (Population × Year), Site (Site × Year), and the triple interaction between them (Year × Population × Site) on resin duct number and density are also shown. Year was considered a repeated measure within individual trees. Degrees of freedom in the numerator and denominator (*NumDf* and *DenDf respectively*), *F*-ratios (*F*) and associated p-values (p) are shown. Significant p-values (p < 0.05) are highlighted in bold. N = 174 trees were analysed across a 31-year period.

Resin duct number in the total ring and the earlywood (but not in the latewood) significantly varied among test sites (Table 1) being higher in RIO than in CAB (Fig. S3a). Resin duct density in the earlywood, latewood and the total ring significantly varied between test sites (Table 1). Pine trees growing in RIO exhibited significantly greater resin duct density in the total ring and earlywood than trees growing in CAB (Fig. S3b). However, pine trees growing in CAB exhibited significantly greater resin duct density in the latewood than trees growing in RIO (Fig. S3b). We did not find evidence of genetic variation in the phenotypic plasticity in resin duct production (no significant Pop × Site effect; Table 1) indicating that responses to local site conditions were similar among populations.

Repeated measures mixed models showed significant interannual variation in resin duct production within subjects along the 31-year period, i.e. within-tree variation or temporal plasticity, as indicated by a significant Year effect when expressed as both resin duct number and density (Table 1, Fig. S4). Moreover, we found significant Pop × Year and Site × Year interactions, indicating that annual patterns of resin duct production, i.e. year-to-year plasticity, was contingent on both the population and the environment (Table 1).

### Correlation between anatomical defences and climate at the population’s origin

We did not find any significant correlation between resin duct production and any of the climate indices at the population’s origin (Table S4).

### Trade-offs between growth and defences

**Figure 2:**
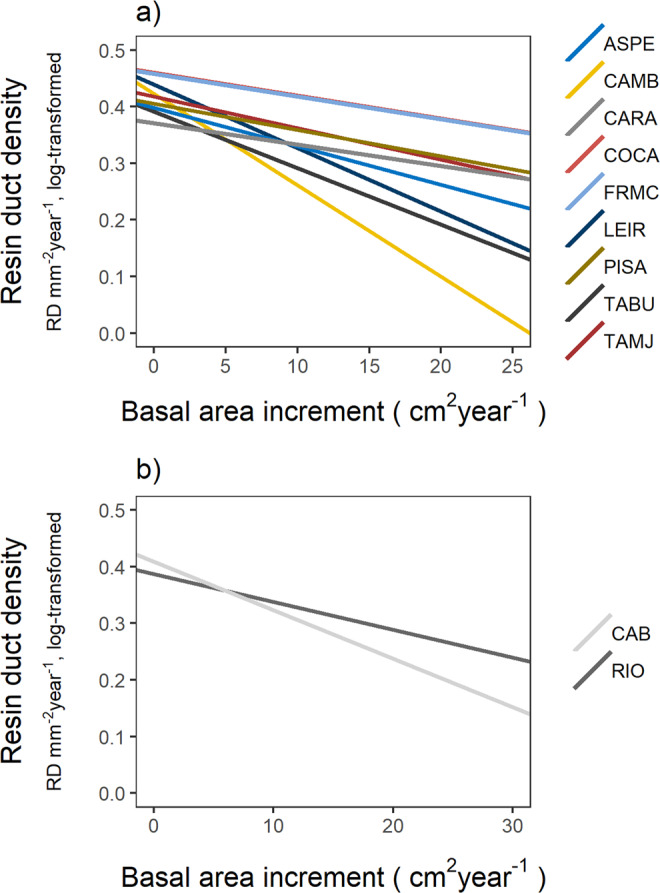
Negative relationship between annual basal area increment (BAI) and annual resin duct density. (**a**) Population effect on the covariation between both traits in *Pinus pinaster* trees from nine populations. Each line represents the linear relationship for each population. (**b**) Site effect (non-significant) on the covariation between both traits in *Pinus pinaster* trees growing in two test sites, Cabañeros (CAB) and Riofrío (RIO). Each line represents the linear relationship for each site. See population codes in Table S1.

We did not find a significant association between growth and anatomical defences at the population level (*r* = −0.39, *p* = 0.31, *N* = 9; Fig. S5). Contrarily, the ANCOVA analysis revealed that BAI had a significant negative effect on resin duct density across years (Table 2; Fig. 2a,b) indicating a physiological trade-off between growth and anatomical defences at the phenotypic level. Furthermore, we found a significant effect of BAI × Pop interaction, revealing differences in the slope parameter among populations (Table 2). This result indicates that the strength of the relationship between annual BAI and annual resin duct density markedly differed among pine populations (Fig. 2a). More negative slope values were indicative of a stronger negative association between resin duct density and BAI. The slope ranged between −0.003 in COCA and −0.02 in CAMB. Finally, we did not find evidence of plastic variation in the expression of the growth-defence trade-off as shown by the non-significant effect of BAI × Site (Table 2; Fig. 2b).

**Table 2:** Results of the ANCOVA analysis showing the covariation of resin duct density (RD year^−1^ mm^−2^) with basal area increment (BAI – cm^2^ year^−1^) in trees from nine *Pinus pinaster* populations planted in two test sites.

	*NumDF, DenDf*	F	P
Basal Area Increment (BAI)	1,1899	59.50	**<0.001**
**Variation in BAI effect**			
BAI × Population	8,1908	2.60	**0.0079**
BAI × Site	1,1899	3.73	0.0536
BAI × Population × Site	8,1908	0.93	0.4942

BAI × Population and BAI × Site effects represent the variation among populations and between sites in the slope of the linear relationship between RD density and BAI. Degrees of freedom in the numerator and denominator (*NumDf* and *DenDf* respectively), *F*-ratios (*F*) and associated *p*-values (*p*) are shown. Significant *p*-values (*p* < 0.05) are highlighted in bold. N = 174 trees were analysed across a 31-year period.

### Correlation between growth-defence trade-offs and climate at the population’s origin

**Figure 3:**
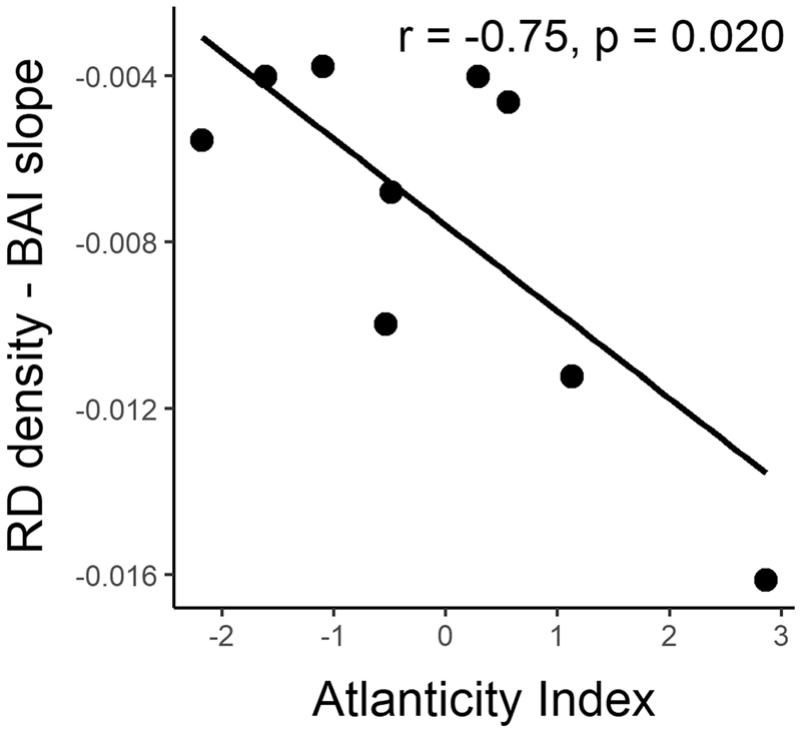
Association between the slope of the linear relationship between basal annual increment (BAI) and resin duct density (RD density) of *Pinus pinaster* trees from nine populations and climate at the population’s origin, particularly with the Atlanticity index. The slope term is an indicator of the strength of expression of growth-defences trade-offs across years for each population (N = 9). More negative values are indicative of more intense growth-defence trade-off expression. Pearson’s r correlation and associated p-values are also shown.

We found a significant correlation between the strength of growth-defence trade-off quantified as the slope value, and the climate at the population’s origin. Specifically, the slope parameter of the growth-defence trade-off for each population was significantly and negatively correlated with the climate index of Atlanticity (Fig. 3). Accordingly, Atlantic populations characterized by more humid conditions, milder temperatures and lower thermic oscillation, showed more negative values of the slope parameter, indicating a stronger negative relationship between RD density and BAI.

## Discussion

### Genetic variation and plastic responses modulate resin duct production

We found that annual resin duct production measured along a 31-year-period largely differed among populations of *P. pinaster*. Population variation in defensive traits can be expected because allocation of resources to defences must be optimized according to long-term evolutionary adaptation to the local biotic and abiotic environments^3,38^. These results are in accordance with previous studies showing population variation in resin duct traits in maritime pine and other conifer species^8,39,40^. Additionally, we also found significant variation among populations in the density and number of resin ducts in the earlywood and latewood. Production of resin ducts during both the early and late growing season is therefore affected by the population genetic background.

Resin duct production also differed between test sites, therefore revealing plasticity to the local environment. Plastic responses in defensive traits to nutrient availability or drought are known to occur in conifers^10,11,28,41,42^. Such responses are necessary to optimize resource allocation to defences and other life functions according to the environment. Our findings support the prediction that plants growing in growth-prone environments invest less in defences^5,26,27^, as trees in the most favourable site, Cabañeros, produced less resin ducts than trees in Riofrío. Trees in Cabañeros, however, showed higher resin duct density in the latewood, although no differences between sites in resin duct number were found in this ring compartment. These results were probably due to the effect of a lower latewood tissue production in trees growing at Cabañeros, since it is at a higher elevation than Riofrío and a shorter growing season can be expected^43^.

The earlywood and the latewood are ring compartments with different cellular structure and function associated with differential climatic conditions during their development. On the one hand, mild temperatures and high water availability during the early growth season promote the development of wider tracheids and higher growth rates^44,45^. On the other hand, during the late growing season growth declines and high temperatures promote lignification of cell walls^46^. As supported by this and other studies, resin duct production seems to be mainly constrained to the late growth season^47,48^. This result agrees with an increased  defensive allocation when environmental conditions limit growth more than photosynthetic activity^5^.

We found a lack of genetic variation in plasticity across sites, as shown by the no significant Pop × Site interaction, indicating similar comparative population performance in both test sites. Similarly, Rosner and Hannrup^49^ showed that variation in resin duct production was genetically determined in *Picea abies*, and that the ranking of the genotypes was stable in two contrasted environments with no significant G × E effects. Contrary to our findings, Moreira, *et al.*^42^ found that *P. pinaster* half-sibs markedly differed in the phenotypic plasticity of resin duct production in response to soil nutrient availability. Likewise, the genotype ranking in resin duct production changed across environments in *Pinus taeda*^50^. Inconclusive or contradictory results may indicate that G × E effects on resin duct production are species-specific or context-dependent.

Plastic responses in resin ducts were also evidenced by the high interannual variation in the production of resin ducts when expressed as both number and density, reflecting high temporal plasticity. Interannual climatic variation is widely accepted as the main environmental factor driving year-to-year variation in tree ring characteristics^37^. Indeed, previous studies have applied dendrochronological methods to quantify the yearly production of axial resin ducts and have demonstrated that climate conditions drive interannual variation in the expression of resin ducts^11,51^. Moreover, our results suggest that such patterns of temporal plasticity are strongly determined by both the genotype and the local environment (significant Pop × Year and Site × Year interactions). This indicates that trees from different populations or growing under different environmental conditions might differentially respond to interannual climate variation. Further studies should therefore test this hypothesis and specifically assess the variation in the climatic responses of resin ducts across populations.

Our results showed that genetic variation in average annual production of resin ducts along a 31-year-period was not associated with climate at the population’s origin (i.e., no evidence of local adaptation to climate). These findings are not consistent with previous studies reporting an association between population differentiation in resin ducts and climate at the population’s origin. For instance, O’Neill *et al.*^8^ found that resin duct production in populations of a boreal spruce planted in common gardens was positively correlated with aridity and continentality and negatively correlated with temperature at the population’s origin. A recent study has also shown that among population variation in resin duct production in the xylem of *P. pinaster* saplings follows climatic clines^40^. Both ontogeny and phylogeny, among other factors, could matter for identifying patterns of adaptive variation in this anatomical trait.

### Differential expression of growth-defence trade-offs among populations

Trade-offs are a central research topic in evolutionary ecology as they are thought to shape diversity patterns in functional traits among and within species^30^. More specifically, negative correlations between plant growth and defences are of special interest given the costs associated to production of anatomical and chemical defences^28^. In this study we found a strong evidence of a physiological growth-defence trade-off at the phenotypic level, indicated by a negative correlation between annual BAI and annual resin duct density. Our results are in agreement with previous studies reporting negative associations between growth and density of resin ducts in adult pine trees^34,52–54^ and saplings^42^. Different resin duct metrics can, however, reveal opposite associations with growth parameters^52^. For instance, a recent study in pinyon pine found a positive association between growth and the absolute number of resin ducts both at the phenotype and the population levels^55^. This might suggest that as radial growth increases, absolute defence production (e.g. resin duct number) is not favoured in the same magnitude, resulting in a dilution effect and thus lower defence-growth ratio (i.e. resin duct density).

Our results do not support predictions of the RAH when invoked to explain patterns of genetic covariation between growth and defences at the intraspecific level, as those populations that grew more did not produce significantly less resin ducts^3^. Trade-offs may not be detected because its expression can be context dependent and emerge only under particular circumstances or environmental conditions^28^. Patterns of covariation between functional traits within and among populations may also be scale dependent and the result of differential evolutionary forces acting at each level^24,30^. Moreover, if enough variation exists in the expression of trade-off within species, such variation might indeed mask negative genetic correlations at a higher hierarchical level. Our results support this idea, as we found that the expression of trade-offs was not homogeneous but rather highly variable among populations, i.e. there is genetic variation in the expression of trade-offs. The fact that some populations may express a trade-off while others may not, can explain why a negative correlation between BAI and resin duct density at phenotypic level did not result in a significant genetic association between both traits across populations.

Our results also showed that the strength of the growth-defence trade-off was significantly correlated with climate conditions at the population’s origin. This result suggests that growth-defence strategies at the population level are likely shaped by adaptive processes. In particular, Atlantic populations characterized by milder temperature conditions and higher precipitation showed a stronger negative association between BAI and resin duct density. Growth-defence trade-offs are more likely to emerge under limiting environmental conditions determining physiological constrains^28,42^. Our results, however, indicate that populations that evolved under more favourable growth conditions (lower thermic oscillation, higher precipitation, milder temperature) showed stronger physiological defensive constraints. This can be explained by the fact that such populations were the most dissimilar in climate conditions to the sites where the trees were planted in central Spain. Indeed, Atlantic populations, which generally show higher intrinsic growth rates (i.e. growth potential), grew less in the test sites^56^. Accordingly, maladaptation to new environmental conditions may promote allocation constrains between growth and defence and affect the performance of populations in the near future. Altogether, our results indicate that growth-defence trade-offs are not only contingent on the environment where plants are growing, but also on their evolutionary context.

Exploring adaptive patterns in functional traits allows us to understand how evolutionary forces acted in the past. Such understanding is required to anticipate population responses to changing biotic and abiotic conditions. Here we found no evidence of patterns of climatic adaptation in resin duct production, although previous studies in the same species observed that population differentiation in other resin duct characteristics follow climate gradients. Our results suggest that defensive investment is not only genetically determined but rather highly contingent on plastic responses to local (spatial) and annual (temporal) environmental variation. Moreover, we found that the expression of growth-defence trade-offs is associated with environmental gradients and likely the result of adaptive processes. Dendrochronological methods allowed us to capture interannual variation in growth and defences during a long time period, ultimately leading to a robust assessment of intraspecific patterns of growth-defence trade-offs. Our results help to understand the evolutionary basis of the allocation compromises between growth and defences and to fill pivotal knowledge gaps in plant defence research.

## Methods

### Study species

Maritime pine (*Pinus pinaster* Ait.) is distributed through the Western Mediterranean basin, from south-western Europe to northern Africa (Fig. 1), occupying heterogeneous habitats ranging from mesic and mild coastal climates to dry and extreme inland climates^57^. As result of its migration patterns from several postglacial refugia and its singular demographic history, *P. pinaster* has now a fragmented distribution with a marked and geographically structured genetic variation^58,59^, and many small populations well differentiated in a number of phenotypic traits^60–62^. These characteristics make *P. pinaster* an appropriate model to explore the individual and interactive effects of genetic factors and plastic responses on functional traits.

### Experimental design

To explore population variation and phenotypic plasticity in resin duct production across environments, we studied two replicated common gardens separated by 30 km, one located at Cabañeros (CAB) and other at Riofrío (RIO), Ciudad Real province, Central Spain (Fig. 1, Table S1). Each test site included trees from 52 populations spread across the distribution range of the species. Test sites were stablished in 1967 following a complete block design, with four blocks of 16 trees per population separated by 2.5 m. Both were characterized by inland Mediterranean climates but strongly differed in topography and soil conditions^56^. Mean annual temperature in RIO was almost 2 °C higher than in CAB (Table S1). RIO was located on a mid-hill with irregular topography whereas CAB was located on a top-hill and was characterised by a flat topography. The soil in CAB had higher content in organic carbon (165 vs 135 organic carbon content per mile) and lower percentage of coarse fragments (19 vs 25% fragments >2 mm) in comparison with the soil in RIO (Worldclim SoilGrids database)^63^. Better soil conditions and milder temperatures in CAB resulted in higher tree survival (90 vs 70% at tree age 18) and growth rate (11.1 vs 9.9 m mean tree height at age 32) in comparison with RIO^56,64^. For the current study we selected a subset of nine populations from seven genetic clusters^59^, covering a wide environmental and geographical variation within the natural distribution range of the species (Fig. 1, Table S1).

### Sampling and dendrochronological procedures

Ten trees from each population and test site were sampled, with the exception of three populations in RIO, for which sample size was 5, 7 and 12 trees (N = 174) (Table S2). Sampling was performed in 2011, when trees were 44 years old. Three or four wood cores per tree were taken at breast height with a 5-mm wide Pressler increment borer to account for within tree variation (Table S2). Cores were glued on wooden mounts and sanded with progressively finer grit size of sandpaper until tree rings and resin ducts were clearly visible. Tree-ring series were visually crossdated assigning calendar years to each tree ring. Width of within-ring compartments, earlywood and latewood, were measured separately with a precision of 0.001 mm using a binocular microscope coupled to a measuring device (Velmex Inc., Bloomfield NY, USA). Width of the total ring was calculated on a yearly basis as the sum of earlywood and latewood widths. Crossdating quality and accuracy was statistically validated using COFECHA software^65^. Annual basal area increment (BAI) was obtained from ring width measurements assuming circularity of the ring sections^66^.

### Anatomical defences

Resin duct records were taken for the period 1980–2010 (31-year-period). In each wood core, annual resin duct number was quantified year-by-year with a binocular microscope separately in the earlywood, the latewood and total ring and expressed as the number of resin ducts per year (RD year^−1^). Annual resin duct density was calculated as the number divided by the corresponding area of tissue (earlywood, latewood and the total ring) and expressed as the number of resin ducts per unit of area and year (RD mm^−2^ year^−1^). Tissue area was computed on the 5-mm wide wood cores assuming squared sections. Values at the tree level were obtained by averaging annual resin duct number and density in the earlywood, the latewood and the total ring, across the 3–4 cores per tree.

### Climate data

To explore the relationship between anatomical defences and the climate at the population’s origin, several climatic variables at the origin of each population were obtained from different climate models. Climate of the five Spanish populations (ASPE, TABU, CAMB, COCA, and CARA) was obtained from a regional climate model^67^ which is known to be highly accurate because it takes into consideration a denser network of meteorological stations^58^. Climate of the remaining four populations (PISA, FRMC, LEIR and TAMJ) was obtained from the publicly available CRU TS 4.01 data set, Climate Explorer of the Royal Netherlands Meteorological Institute (http://climexp.knmi.nl)^68^. Monthly time series of mean temperature, maximum temperature in the warmest month, minimum temperature in the coldest month, temperature seasonality, annual and summer precipitations, and average number of frost days per year were retrieved from these models for each population and year in the period 1950–2000, and averaged across years. In order to utilize all climate information without inflating type I error through multiple testing, all climatic variables were summarized into two main principal components (hereafter ‘climate indices’) using a principal component analyses. Climate index 1 (Atlanticity Index) explained 46.5% of the variance and was positively related to warmer minimal temperatures and high annual precipitation and inversely related to temperature seasonality, resulting in an axis of variation from Continental and Mediterranean climates (low values of the climate index) to Atlantic climates (high values) (Fig. S1). Climate Index 2 explained 37.2% of the total variance and was positively related to higher mean temperatures and negatively related to frost frequency, suggesting a thermal gradient from cold to warm conditions (Fig. S1) at low and high values of the climate index, respectively.

### Statistical analysis

Annual production of resin ducts (number and density) was analysed by linear mixed models for repeated measures using the *lmer* function from the *LmerTest* package in *R*^69^. The response variables were log-transformed to achieve normality. To assess the individual and interactive effects of genetic variation and phenotypic plasticity on resin duct production across subjects, Population (Pop), test site (Site) and their interaction (Pop × Site) were included in the model as fixed factors. To account for repeated measures within the same individuals, the Year and its interaction with Pop and Site were included as fixed factors while the subject, i.e. tree identity, was included as a random factor. Such within subject analysis allowed us to assess inter-annual patterns in resin duct production and their variation due to genetic or environmental effects. Block within site (B[Site]) and its interaction with population (B[Site] × Pop) were first included in the model as random factors but they did not improve the fit of the model, i.e. AIC values. These terms were therefore removed from all models. Least squared means for each population were extracted from the mixed model using the *lsmeans* function in R^70^ to be used for further analysis.

Pearson’s correlation analysis between BAI and resin duct density in the total ring was used to test for a negative association between growth and defences at the population level. Resin duct density in the total ring was used because it represents a standardized measure of production of anatomical defences during the whole growth season. Additionally, we used analysis of covariance (ANCOVA) on yearly records of BAI and resin duct density to test for a trade-off between growth and defences. ANCOVA was used to assess the overall effect of BAI on resin duct density and the homogeneity of such effect among groups, i.e. homogeneity of slopes among populations and test sites. Resin duct density (log-transformed data) in the total ring was analysed using the aforementioned linear mixed model, but including the annual BAI as a covariate. The interaction between BAI and the population (BAI × Pop) and BAI and the site (BAI × Site) were included in the model to test for differences among populations and test sites in the slope of the linear relationship between resin duct density and BAI. In case of a significant BAI × Pop interaction, the slope parameter for each population was used as a quantitative indicator of such relationship, i.e., the strength of the observed trade-off for each population, for further analysis.

To assess whether genetic variation in resin duct production has a climate-driven adaptive origin, population least square means of resin duct number and density in the earlywood, the latewood and the total ring were correlated with climate at the population’s origin by using parametric correlation analysis. Pearson’s correlation coefficients were calculated for associations between resin duct characteristics and the climatic indices summarizing climate conditions at the population’s origin.

The slope for the relationship between resin duct density in the total ring and annual BAI for each population was also correlated with the climatic indices summarizing climate conditions at the population’s origin to test for a potential adaptive origin in the expression of growth-defence trade-offs.

## Data availability

The datasets generated and/or analysed during the current study are available from the corresponding author on request.

## Supplementary information

Supplementary Information.
