# Supplementary material for: Climate drives intraspecific differentiation in the expression of growth-defence trade-offs in a long-lived pine species
Source: Sci Rep. 2020 Jun 29;10:10584. doi: 10.1038/s41598-020-67158-4 (PMC7324371; doi:10.1038/s41598-020-67158-4)

**Climate drives intraspecific differentiation in the expression of growth-defence trade-offs in a long-lived pine species**

Carla Vázquez-González^*1^, Luis Sampedro^1^, Vicente Rozas^2,3^ and Rafael Zas^1^

**Supplementary information**

**Supplementary table S1** Population and site characteristics including geographic variables (elevation, latitude and longitude), total annual precipitation (PPT) and annual mean temperature (AMT).

| Site name | Abbreviation | Genetic group^1^ | Elevation  (m) | Latitude | Longitude | PPT  (mm^3^) | AMT  (ºC) |
| --- | --- | --- | --- | --- | --- | --- | --- |
|  |  |  |  |  |  |  |  |
| Trial test site |  |  |  |  |  |  |  |
| Cabañeros | CAB |  | 1023 | 39.3960 | -4.4750 | 796.00 | 12.4 |
| Riofrio | RIO |  | 725 | 39.1340 | -4.5360 | 719.00 | 14.2 |
|  |  |  |  |  |  |  |  |
| Populations |  |  |  |  |  |  |  |
| Arenas de Sampedro | ASPE | Central Spain^3^ | 710 | 40.2092 | -5.0583 | 1217.00 | 13.7 |
| Tabuyo | TABU | Atlantic^3^ | 1005 | 42.3144 | -6.2197 | 734.00 | 9.17 |
| Cambados | CAMB | Northern Spain^3^ | 17 | 42.5264 | -8.7697 | 1550.00 | 14.7 |
| Coca | COCA | Central Spain^3^ | 781 | 41.2481 | -4.4931 | 454.00 | 12.3 |
| Pisa | PISA | Northern Italy^2^ | 100 | 43.7172 | 10.3839 | 859.57 | 13.9 |
| Massif Central | FRMC | Atlantic France^2^ | 400 | 45.0006 | 3.7503 | 839.46 | 13.1 |
| Tamjout | TAMJ | Western Africa^2^ | 1600 | 33.8669 | -4.0333 | 335.86 | 14.2 |
| Leiría | LEIR | Atlantic^2^ | 60 | 39.7506 | -8.9169 | 934.18 | 15.6 |
| Caravaca | CARA | Eastern Spain^3^ | 1104 | 38.1450 | -1.9839 | 481.00 | 13 |
|  |  |  |  |  |  |  |  |

^1^ Genetic groups according to Bucci et al. (2007)

^2^ Climatic data obtained from the CRU TS 4.01 data set for the period 1980-2010

^3^ Climatic data obtained from regional models in Gonzalo (2007)

|  | Cabañeros | | Riofrío | |
| --- | --- | --- | --- | --- |
| Population | Nº of trees | Nº of cores | Nº of trees | Nº of Cores |
| ASPE | 10 | 40 | 10 | 40 |
| TABU | 10 | 40 | 10 | 40 |
| CAMB | 10 | 39 | 10 | 40 |
| COCA | 10 | 39 | 5 | 20 |
| PISA | 10 | 40 | 10 | 40 |
| FRMC | 10 | 39 | 7 | 28 |
| TAMJ | 10 | 40 | 12 | 48 |
| LEIR | 10 | 40 | 10 | 40 |
| CARA | 10 | 40 | 10 | 40 |
| Totals | 90 | 357 | 84 | 336 |

**Supplementary table S2** Number of trees and cores sampled in each population in both test sites, Cabañeros and Riofrío.

**Supplementary figure S1.** Principal component analysis of climate data showing the spread of the 9 maritime pine populations (grey dots) and the test sites (black dots) along the two first principal components (climate indices), and the loadings of the climate variables on these climate indices (grey arrows). Climate index 1 (PC1, 46.5% of variance) was a proxy of Continental and Mediterranean climates at low values and Atlantic climates at high values. Climate index 2 (PC2, 37.2% of variance) reflected a thermal gradient from cold (low values) to warm (high values) temperatures.


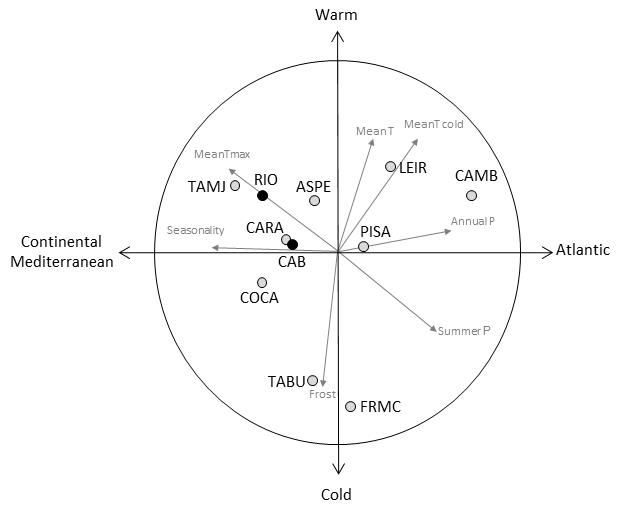


**Supplementary table S3** Mean, minimum (Min.) and maximum (Max.) value of average annual resin duct number (RD number – RD year ^-1^) and density (RD density – RD mm ^-2^ year ^-1^) in the earlywood, the latewood and the total ring of 174 Pinus pinaster trees. Standard deviations (SD) and standard errors (SE) are also shown.

|  | **Total Ring** | | **Earlywood** | | **Latewood** | |
| --- | --- | --- | --- | --- | --- | --- |
| *N= 174* | *RD number* | *RD density* | *RD number* | *RD density* | *RD number* | *RD density* |
| Mean | 4.18 | 0.51 | 2.00 | 0.22 | 2.19 | 1.49 |
| Min. | 1.73 | 0.32 | 0.26 | 0.04 | 0.91 | 0.73 |
| Max. | 7.63 | 0.82 | 5.13 | 0.51 | 4.07 | 2.67 |
| SD | 1.15 | 0.09 | 1.02 | 0.09 | 0.54 | 0.38 |
| SE | 0.09 | 0.006 | 0.08 | 0.006 | 0.04 | 0.002 |
|  |  |  |  |  |  |  |

**Supplementary figure S2** (a) Resin duct number (RD number) and (b) resin duct density (RD density) in the earlywood and latewood and in the total ring of nine Pinus pinaster populations. Bars represent least squared means ± SE computed from the corresponding repeated measures analysis. N =174 trees were analysed across a 31-year period. See population codes in Table S1.

*
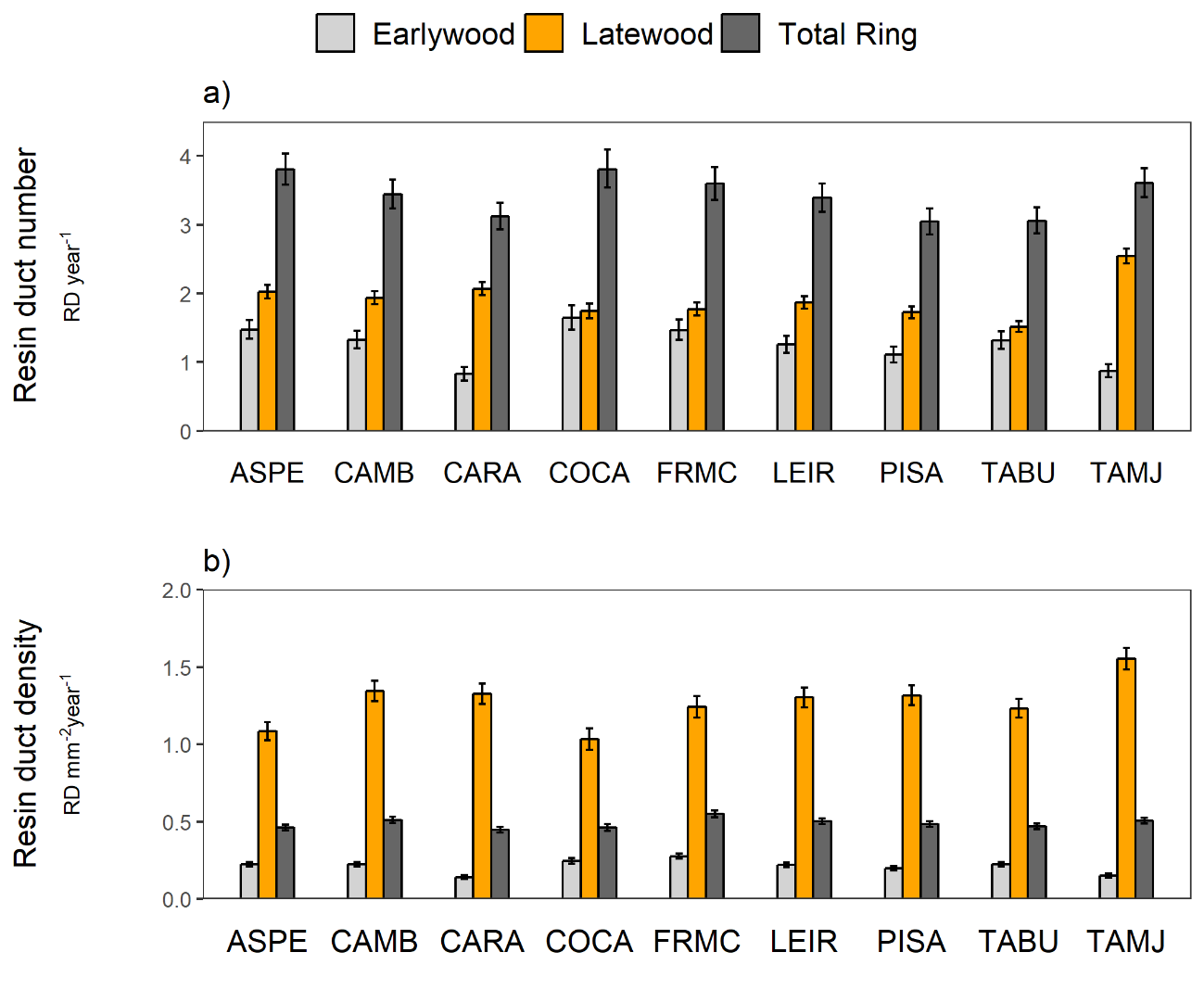
*

**Supplementary figure S3** (a) Resin duct number and (b) density in the earlywood, latewood and the total ring of Pinus pinaster trees growing in two common gardens located in Cabañeros and Riofrío. Bars represent least squared means ± SE computed from the corresponding repeated measures analysis. N =174 trees were analysed across a 31-year period.


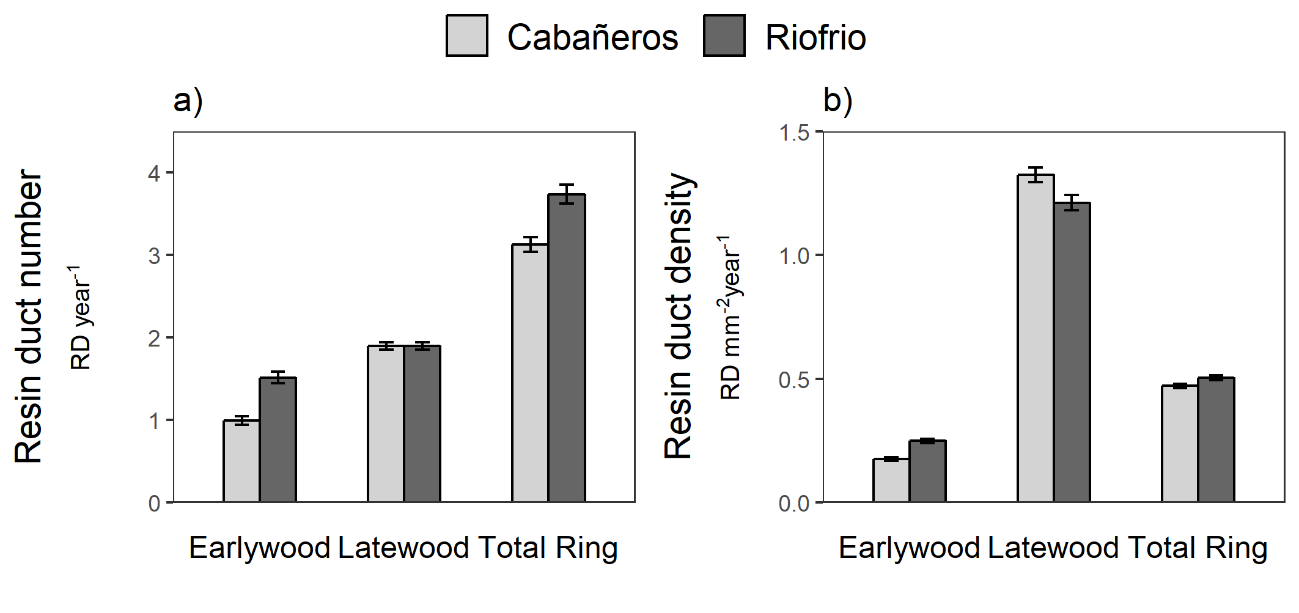


**Supplementary figure S4** Resin duct number (RD number) and density (RD density) in the earlywood, latewood and the total ring of Pinus pinaster trees growing in Cabañeros and Riofrío from 1980 to 2011 averaged across populations. N =174 trees were analysed across a 31-year period.


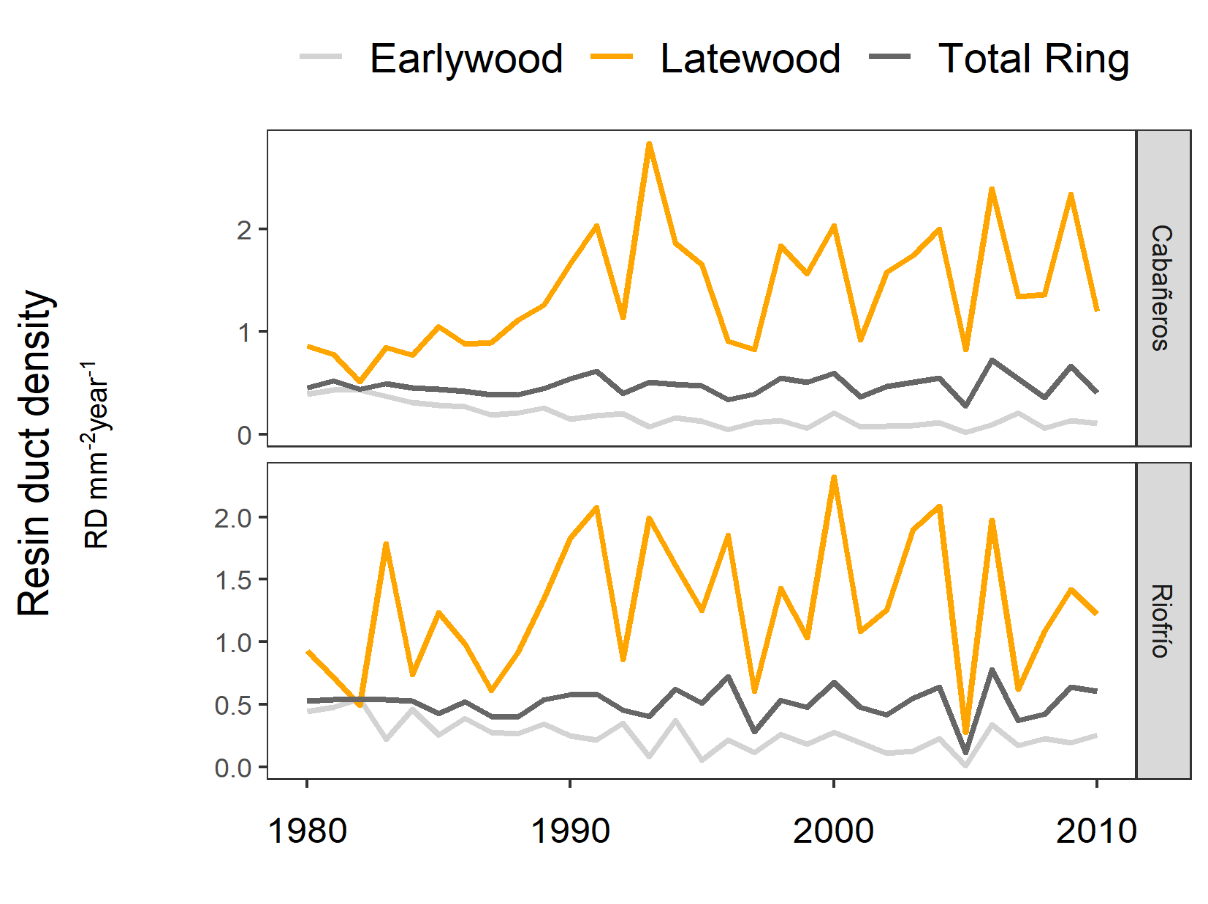

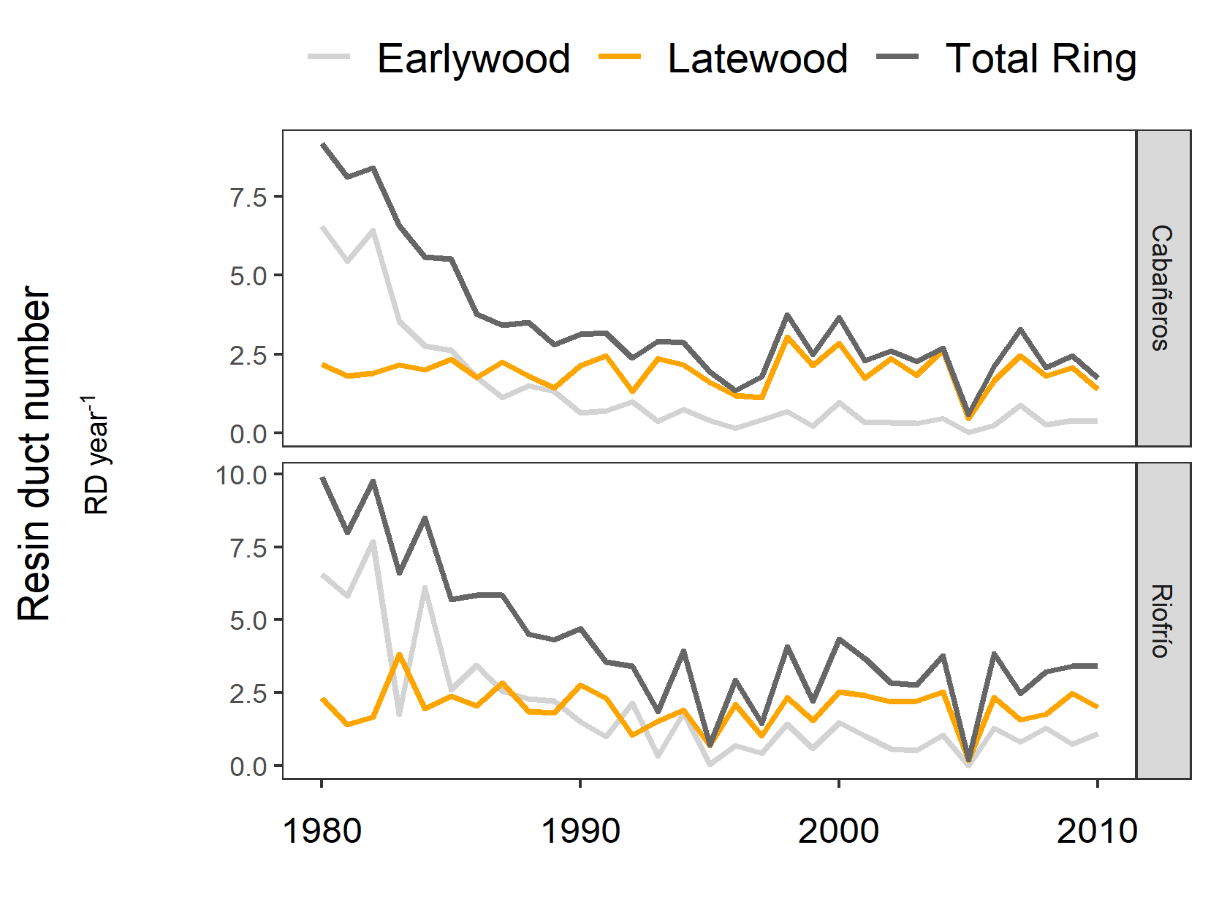


**Supplementary table S4** Correlation coefficients for the association between resin duct characteristics of each *Pinus pinaster* population and climate indices indicative of climatic conditions at the population’s origin. Pearson’s r correlation coefficients (Pearson’s r) and associated probability values (p-values) are shown (N = 9)

|  | ***PC1 (Atlanticity index)*** | | | ***PC2 (temperature index)*** | | |
| --- | --- | --- | --- | --- | --- | --- |
|  | Pearson’s r | | p-value | Pearson’s r | | p-value |
| ***Resin duct number*** |  |  | |  |  | |
| Earlywood | 0.21 | 0.589 | | -0.37 | 0.333 | |
| Latewood | -0.35 | 0.359 | | 0.62 | 0.075 | |
| Total ring | -0.22 | 0.566 | | 0.15 | 0.685 | |
| **Resin duct density** |  |  | |  |  | |
| Earlywood | 0.38 | 0.312 | | -0.523 | 0.148 | |
| Latewood | 0.04 | 0.907 | | 0.32 | 0.393 | |
| Total ring | 0.44 | 0.234 | | -0.20 | 0.601 | |
|  |  |  | |  |  | |

**Supplementary figure S5** Association between annual resin duct production (RD density) and basal area increment (BAI) among 9 *Pinus pinaster* populations (N = 9). Each point represents least squared population means. Pearson’s r correlation and associated p-values are shown.


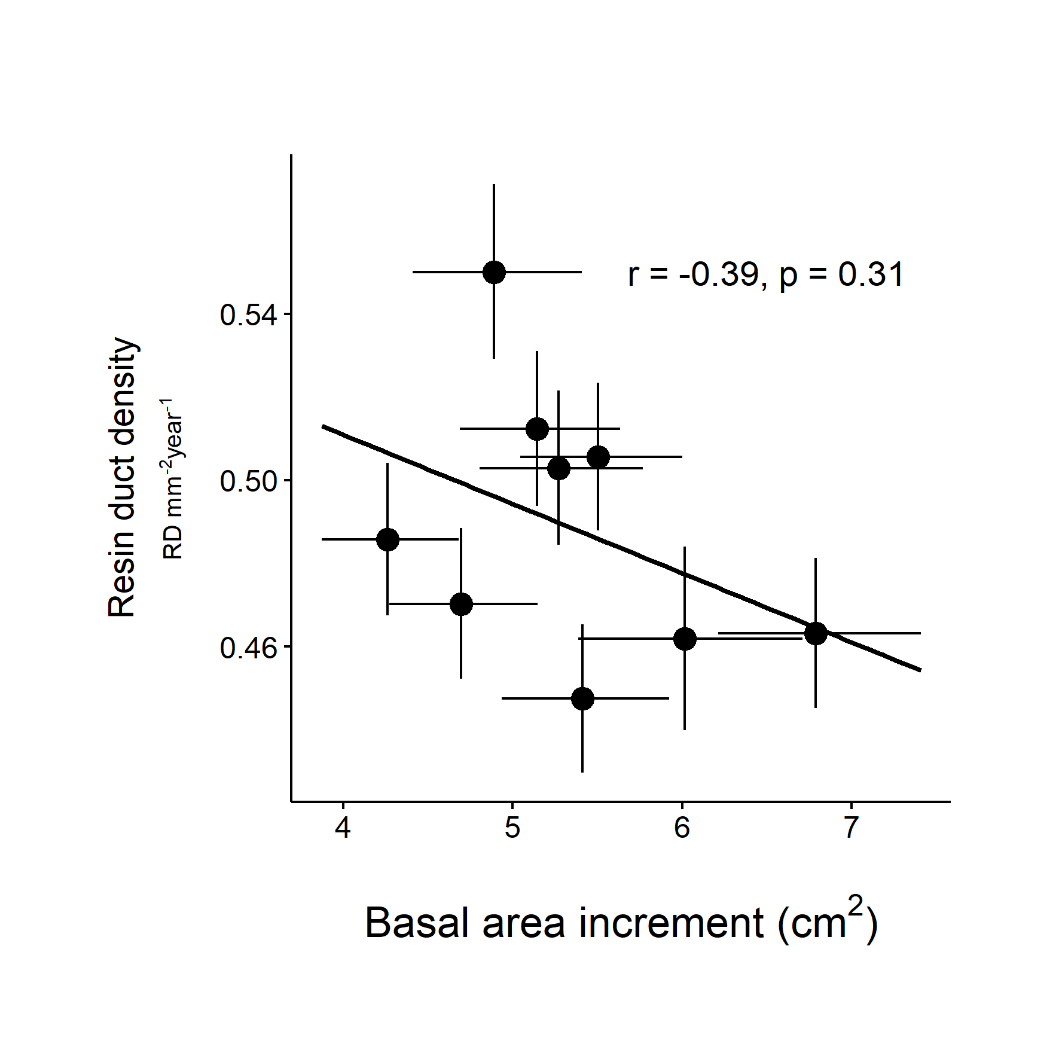

Supplement: Supplementary file 1 — Supplementary Information. [file 41598_2020_67158_MOESM1_ESM.docx]
